# Individual tree traits shape insect and disease damage on oak in a climate‐matching tree diversity experiment

**DOI:** 10.1002/ece3.5357

**Published:** 2019-07-17

**Authors:** Elsa Field, Karsten Schönrogge, Nadia Barsoum, Andrew Hector, Melanie Gibbs

**Affiliations:** ^1^ Department of Plant Sciences University of Oxford Oxford UK; ^2^ Centre for Ecology & Hydrology Crowmarsh Gifford Wallingford UK; ^3^ Forest Research Farnham Surrey UK

**Keywords:** associational resistance, *Erysiphe alphitoides*, mixed stands, oak powdery mildew, plant apparency, plant vigor, plant–herbivore interactions, *Quercus robur*, tree diversity

## Abstract

Diversifying planted forests by increasing genetic and species diversity is often promoted as a method to improve forest resilience to climate change and reduce pest and pathogen damage. In this study, we used a young tree diversity experiment replicated at two sites in the UK to study the impacts of tree diversity and tree provenance (geographic origin) on the oak (*Quercus robur*) insect herbivore community and a specialist biotrophic pathogen, oak powdery mildew. Local UK, French, and Italian provenances were planted in monocultures, provenance mixtures, and species mixes, allowing us to test whether: (a) local and nonlocal provenances differ in their insect herbivore and pathogen communities, and (b) admixing trees leads to associational effects on insect herbivore and pathogen damage. Tree diversity had variable impacts on foliar organisms across sites and years, suggesting that diversity effects can be highly dependent on environmental context. Provenance identity impacted upon both herbivores and powdery mildew, but we did not find consistent support for the local adaptation hypothesis for any group of organisms studied. Independent of provenance, we found tree vigor traits (shoot length, tree height) and tree apparency (the height of focal trees relative to their surroundings) were consistent positive predictors of powdery mildew and insect herbivory. *Synthesis*. Our results have implications for understanding the complex interplay between tree identity and diversity in determining pest damage, and show that tree traits, partially influenced by tree genotype, can be important drivers of tree pest and pathogen loads.

## 1 INTRODUCTION

Much ecological research in recent decades has shown that plant species richness has substantial effects on different aspects of ecosystem functioning (Isbell et al., 2017). For instance, increasing plant species richness has been shown to positively benefit productivity (Benneter, Forrester, Bouriaud, Dormann, & Bauhus, 2018; Pretzsch et al., 2013; Tilman, Wedin, & Knops, 1996), temporal stability (Morin, Fahse, de Mazancourt, Scherer‐Lorenzen, & Bugmann, 2014) and resistance to natural disturbances and climate change (Isbell et al., 2015; Jactel et al., 2017). Diversifying production forests using both species and genetic diversity can result in an economic insurance effect, such that if one crop fails due to adverse environmental changes, forest managers have alternatives in the same plantation to ensure profit (Jactel et al., 2017). In addition, mixed‐species forests may also be intrinsically more resistant to pest and disease outbreaks. Meta‐analyses have shown a trend toward reduced insect damage in tree species mixtures compared to monocultures (Castagneyrol, Jactel, Vacher, Brockerhoff, & Koricheva, 2014; Jactel & Brockerhoff, 2007), and the same has been reported for forest pathogens (Hantsch et al., 2014; Pautasso, Holdenrieder, & Stenlid, 2005).

The reduction of pest and disease damage in mixtures may be due to a pure dilution effect, where lower host abundance in a given area reduces pest abundance on susceptible hosts, as pest species are unable to build up to epidemic levels (Civitello et al., 2015). Additionally, there may be associational effects, relating to the impact of planting nonhost neighbor trees around a focal tree (Barbosa et al., 2009). In associational resistance, a focal host experiences lower pest damage in mixtures compared to monocultures, while associational susceptibility describes the opposite (Barbosa et al., 2009). Mechanisms proposed to underpin associational effects are diverse. Direct effects of nonhost neighbor trees may cause associational resistance by influencing the microclimate of plots with knock‐on impacts on insects (Muiruri & Koricheva, 2017), or by interfering with olfactory cues used by pests to find hosts (Jactel, Birgersson, Andersson, & Schlyter, 2011). Tree species diversity may also modify plant apparency, which can be defined the height of focal trees relative to their surroundings, and has been shown to be an important predictor of insect herbivore loads on individual trees (Castagneyrol, Giffard, Péré, & Jactel, 2013; Régolini et al., 2014). Moreover, complex indirect effects of plant neighborhood identity on insect herbivory and pathogens may be mediated via modifications to plant traits, including foliar nutritional quality and defenses against herbivory (Castagneyrol et al., 2017; Forey et al., 2016).

Genetic diversification of forests may also be encouraged as a climate change adaptation strategy (Bucharova et al., 2018). In forestry, tree provenance is defined as the geographic location of the original stand from which seed or cuttings were taken (Hubert & Cottrell, 2007). The local adaptation hypothesis predicts that local provenance trees will be optimally adapted for local environmental conditions (Bucharova et al., 2017). Assisted migration of provenances from areas predicted to meet the future climate of a region (“climate matching”) provides an alternative to planting local material that may be favorable if climate change outpaces the speed of adaptation of local provenances (Broadmeadow, Ray, & Samuel, 2005; Ennos, Cottrell, Hall, & O'Brien, 2019; Marris, 2009). Climate‐matched provenances may also experience growth benefits if they escape some damage by local pest and disease populations, which can be co‐adapted to local hosts through tight phenological synchrony (van Asch & Visser, 2007; Egan & Ott, 2007) and co‐adaptation to functional traits such as plant defenses (Pearse & Hipp, 2009). However, non‐native provenances may also experience stressful growing conditions under current local climates, leading to reduced tree vigor. Plant stress generally has a negative effect on the performance of primary foliar insect herbivores and biotrophic pathogens, while plant vigor can have the opposite effect (Huberty & Denno, 2004; Jactel et al., 2012). Despite the potential importance of introduced provenances as a future‐proofing strategy against climate change, few experimental studies exist comparing the growth characteristics and pest resistance of local versus climate‐matched provenances under current climatic conditions (Barsoum, 2015; Broadmeadow et al., 2005).

While provenance identity and tree diversity can both shape associated pest communities (Dickson & Whitham, 1996; Wimp, Martinsen, Floate, Bangert, & Whitham, 2005), the simultaneous impacts of diversifying both the genetic and species composition of forest stands have rarely been tested. Moreover, tree diversity effects on pathogens have been less frequently investigated than for insect pests (Hantsch et al., 2014; Jactel & Brockerhoff, 2007; Pearse & Hipp, 2009). Even fewer studies have considered diversity effects on both pests and pathogens together, despite the potential for multitrophic interactions which could impact upon associational effects (Schuldt et al., 2017). For instance, initial damage to a plant by insect herbivores or pathogens can have both a reciprocal and antagonistic effect on subsequent plant attackers, via cross‐talk between plant defense signaling pathways that are induced following attack (Moreira, Abdala‐Roberts, & Castagneyrol, 2018).

Here, we tested the impacts of varying both tree species and genetic diversity on oak (*Q. robur)* foliar insect herbivores, oak powdery mildew and the interaction between the two groups. We used a tree diversity experiment replicated at two sites in the UK, in which local and climate‐matched oak provenances are planted as monocultures or in mixtures of provenances and species. There is increasing evidence that tree diversity effects on pest organisms are context dependent and can be modulated by abiotic conditions such as temperature and water availability impacting upon plant growth and functional traits (Castagneyrol, Moreira, & Jactel, 2018; Kambach, Kühn, Castagneyrol, & Bruelheide, 2016; Walter et al., 2012). By studying replicate trials between years (2 years of data from one site) and between trials, we looked for consistent impacts of provenance identity and tree diversity across space and time. By measuring herbivory by three insect guilds differing in their life history strategies and powdery mildew infection, on the same trees, we were able to look for interactions between insects and pathogens interacting with tree diversity. We suspected the presence of interactions between the two groups as oak powdery mildew has been reported to benefit from insect herbivory, where early season insect defoliation promotes an increase in secondary bud flushes (“lammas shoots”), providing more susceptible leaf material for mildew infection (Marçais & Desprez‐Loustau, 2014).

We tested the following hypotheses:

1. Local and nonlocal provenances would differ in their tree phenotypic traits. Phenotypic traits measured were those with a known impact on insect herbivores and foliar pathogens: plant vigor (tree height and shoot length, both commonly used proxys of vigor (Flaherty & Quiring, 2008; Gripenberg, Ovaskainen, Morriën, & Roslin, 2008)) and the number of lammas shoots produced (as a predictor of powdery mildew infection).
2. Insect herbivore abundance and powdery mildew infection would be highest on local provenances (local adaptation hypothesis).
3. Associational resistance to both insect herbivores and powdery mildew would occur in mixed species plots compared to single species plots.
4. Differences in provenance susceptibility to insect herbivores and powdery mildew could lead to associational effects in mixed provenance plots, compared to provenance monocultures.
5. Both tree vigor, and tree apparency, would be significant positive drivers of insect herbivory and powdery mildew infection.
6. Insect herbivory and oak powdery mildew infection would be positively associated with the same trees.

Our results emphasize the importance of considering tree identity as well as diversity setting in the design of mixed forests, and illustrate how environmental context can affect plant growth, with impacts on forest pests and pathogens.

## 2 MATERIALS AND METHODS

### 2.1 Experimental field sites

The study took place at the Climate Match tree diversity experiment planted in 2011 at two sites in the UK. One site was established in South East England at Hucking, Kent (51°17'47.5"N 0°37'58.2"E), the other in the East Midlands at Hartshorne, Derbyshire (52°47'41.1"N, 1°30'58.8"W).

**Figure 1:**
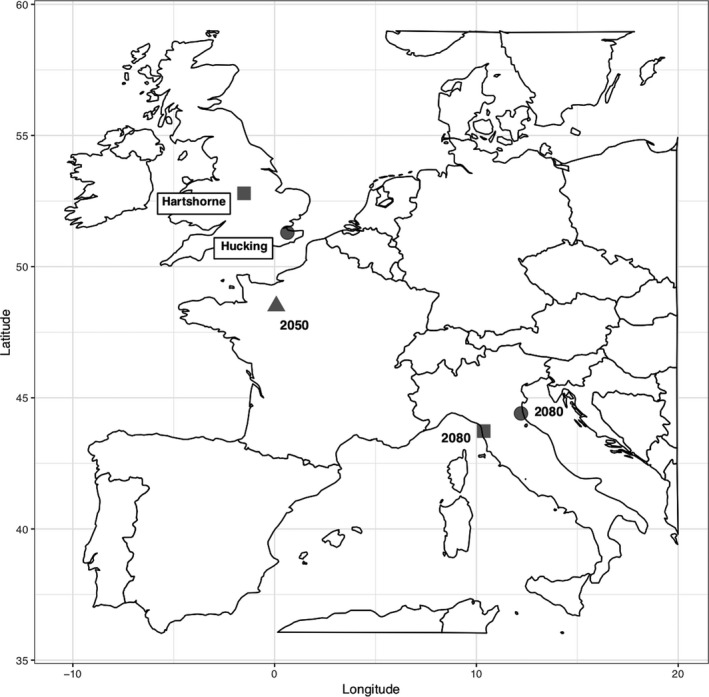
Location of UK Climate Match experiment sites, Hucking in Kent (black circle) and Hartshorne in Derbyshire (black square), and sources of “climate‐matched” oak provenances. At Hucking, local provenance material was from Kingsnorth, Kent, at Hartshorne it was UK 404 provenance. The same provenance (QR0100 North‐West) was planted from France at both sites to represent a 2050 climate match (black triangle). At Hucking, the 2080 climate‐matched provenance planted was from the Ravenna region of Italy (black circle), while at Hartshorne it was from San Rossore region of Italy, Tuscany (black square)

Four broadleaved species of varying provenance were planted: wild cherry (*Prunus avium*)*,* sweet chestnut (*Castanea sativa*), ash (*Fraxinus excelsior*), and pedunculate oak (*Q. robur*). UK provenances of the four tree species were sourced from areas delineated as native seed zones (Herbert, Samuel, & Patterson, 1999) while French and Italian provenances were sourced from regions “climate‐matched” to predictions of the UK climate in 2050 and 2080, respectively (Barsoum, 2015; Figure 1). Due to the trial sites being located in different geographic regions of the UK, local and climate‐matched provenances differed between sites for oak, with only the French provenance in common between the two sites (Figure 1).

**Figure 2:**
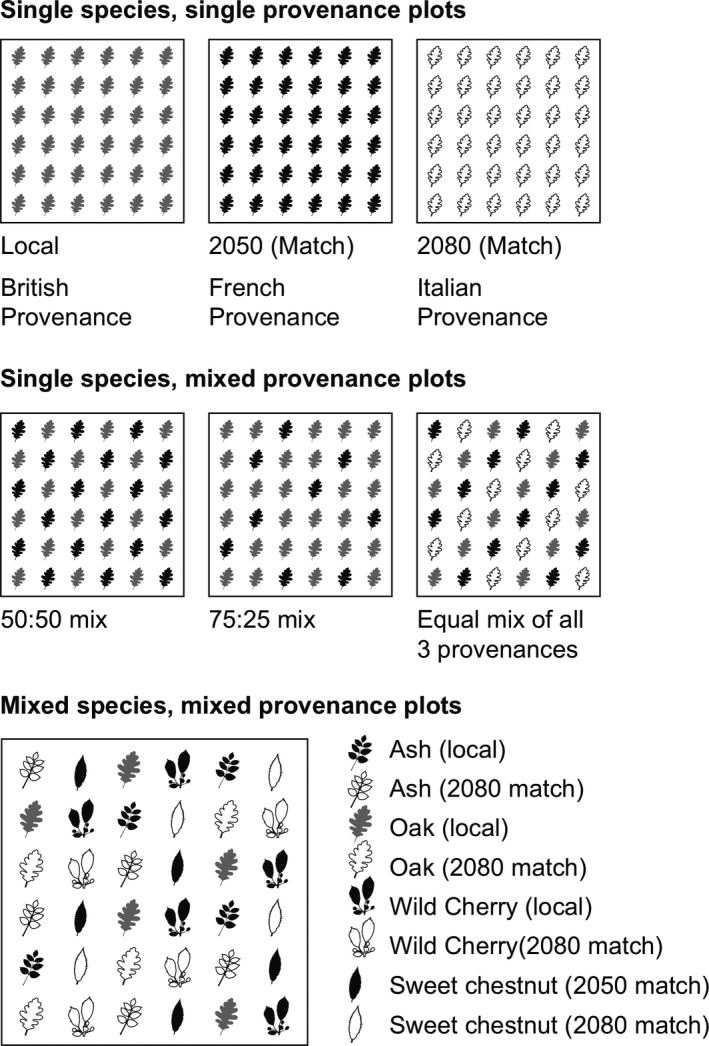
Design of the treatments planted at the Climate Match experiment. Each treatment is replicated at each site in three consecutive experimental blocks, apart from the mixed species plots at the Hartshorne site, where there are replicates in two out of three blocks only, due to site space restrictions

The two trials follow a similar planting design (Figure 2). There are three experimental blocks with treatments replicated once per block for each tree species. We focused only on treatments containing oak: monocultures of Local, French and Italian provenances, 50:50 provenance mixtures (Local:Italian and Local:French), 75:25 provenance mixtures (Local:Italian and Local:French), 33:33:33 provenance mixtures (Local: French: Italian), and mixed species plots (containing Local and Italian provenances of each of the four species). Within mixtures, trees are planted in a regular pattern ensuring different provenances (or species) are mixed with no two trees of the same type next to each other (Figure 2). Provenance monocultures and mixed provenance plots are 12 × 12 m, with 36 trees per plot planted at 4 m^2^ spacing giving six rows of trees of six trees each. Mixed species, mixed provenance plots are 36 × 32 m, containing 288 trees in 16 rows of 18 trees each. Trials were planted using 2‐year‐old saplings. For more information on the trial design and establishment, see Barsoum (2015). The experiment is part of TreeDivNet, a global network of tree diversity experiments (http://www.treedivnet.ugent.be).

### 2.2 Study system

We studied three insect guilds (“insect herbivores”) on oak (*Quercus robur*): cynipid gallwasps (“gallers”), leaf miners, and leaf manipulators. Gallers and leaf miners were known as specialist herbivores of the genus *Quercus* section Quercus (Hayward & Stone, 2005; Nieukerken & Johansson, 2003), whereas leaf manipulators described free‐feeding herbivores that included both generalist and specialist species. The most abundant gallers were the spangle galls *Neuroterus quercusbaccarum*, *N. numismalis*, and *N. anthracinus*. The most abundant leaf miner species were *Phyllonorycter* sp. and *Stigmella* sp.

We measured infection by the specialist biotrophic pathogen, oak powdery mildew, caused by multiple cryptic species of *Erysiphe* with different phenological niches: *E. quercicola* infects both buds and leaves, and *E. alphitoides* infects leaves (Marçais & Desprez‐Loustau, 2014). Surveying of leaves in August means that we likely scored *E. alphitoides,* believed to be the most common species in the UK (Desprez‐Loustau et al., 2018). However, in the absence of molecular identification, we use the term “powdery mildew” to describe the pathogen.

We observed that in the UK, insect herbivore and powdery mildew damage are temporally and spatially separate. Almost all insect herbivore damage occurs on primary shoot leaves emerging during the first flush in late spring (April–May), while oak powdery mildew infection occurs on leaves on lammas shoots emerging during subsequent bud bursts in June–August, consistent with *E. alphitoides* releasing ascospores from overwintering chasmothecia in early to mid‐summer (Marçais, Kavkova, & Desprez‐Loustau, 2009). Oakleaf susceptibility to powdery mildew declines sharply following bud burst, so that leaves on primary shoots largely remain completely uninfected (Ayres & Edwards, 1982). Thus, we recorded herbivory on primary shoots, and powdery mildew infection on lammas shoots, which can be clearly differentiated by a ring scar separating different flushes.

### 2.3 Tree selection for surveying

At both the Hucking and Hartshorne sites, nine oak trees of each provenance per plot were selected for recording both powdery mildew and herbivory, by starting in the core of the plot and moving outwards. Powdery mildew and herbivory were scored in 2016 only at Hucking and at both Hucking and Hartshorne in 2017. This gave a total of 411 trees sampled at Hartshorne, and 420 and 416 trees sampled at the Hucking site in 2016 and 2017, respectively. The difference in tree numbers at Hucking was due to tree mortality between years.

### 2.4 Lammas shoot scoring

To establish whether provenances differed in the timing of lammas shoot production, in July 2017 we randomly selected three trees per provenance in the core of each plot (a subset of trees later scored for herbivory and mildew). On each tree, we randomly selected five primary shoots at varying heights and all aspects around the tree and recorded the presence of one or more second flush shoots, to produce an index of “lammas shoot availability” per tree (the proportion of five shoots producing lammas shoots). This snapshot of lammas shoot growth reflects relative differences in the abundance of lammas shoots on different trees, when scored at the same time point during summer. This allowed us to test for differences between provenances in the amount of susceptible leaf material for mildew infection available at the start of the infection window (between July–August for oak powdery mildew in the UK).

### 2.5 Oak powdery mildew

In August at each site (in 2016 and 2017 for Hucking, 2017 only for Hartshorne), powdery mildew infection was recorded on 10 lammas (second flush) shoots per tree. Shoots were sampled randomly across a range of heights and from all aspects around the tree. We used a 0–3 scale of infection severity based on the % of total leaf material on the shoot infected by fungal mycelium: 0 (0% infection), 1 (<50% infected), 2 (51%–75% infected), 3 (76%–100% infected). This four‐point scale is similar to those used by other authors scoring this pathogen (Bert, Lasnier, Capdevielle, Dugravot, & Desprez‐Loustau, 2016). We also recorded lammas shoot length (mm) to use as a covariate.

### 2.6 Insect herbivores

In August at each site (in 2016 and 2017 for Hucking, 2017 only for Hartshorne), insect herbivore abundance was recorded on 10 primary shoots per tree. Shoots were sampled randomly across a range of heights and from all aspects around the tree. We recorded insect herbivore abundance by counting the number of leaf mines, galls (on leaves and buds), and incidences of leaf skeletonizers, leaf rollers and leaf webbers for all leaves on a shoot (after Sinclair et al., 2015; Moore, Warrington, & Whittaker, 1991). We also recorded primary shoot length (mm) to use as a covariate.

### 2.7 Tree height and apparency

At the end of the recording period in August 2017, we recorded the height of all sampled trees, as well as 27 randomly chosen additional trees per plot, to calculate an index of tree apparency based on the index used in Castagneyrol, Jactel, and Moreira (2018). This was calculated as:

$$\text{Plant Apparency =}\;\left(\frac{\text{Height Focal tree - Average Height of Plot}}{\text{Average Height of Plot}}\right)\times \text{100}$$

### 2.8 Statistical analysis

All analyses were completed in R version 3.4.3 (R Core Team, 2017).

#### 2.8.1 Phenotypic traits of different provenances

To determine whether provenances differed in tree phenotypic traits, we conducted one‐way ANOVAs by provenance for each trait at each site and year. Tree traits analyzed were as follows: tree height, primary shoot length, lammas shoot length, and lammas shoot availability. As data on insect herbivore abundance and powdery mildew infection was analyzed separately for each site and year, we also split the analysis of tree traits in the same way, to allow comparability between analyses.

#### 2.8.2 Powdery mildew infection and insect herbivore abundance

We used generalized linear models (GLMs) to test for differences in insect herbivore abundance and powdery mildew infection on *Q. robur* trees between provenances and diversity settings. We favoured GLMs over Mixed Effect Models as the factor describing spatial autocorrelation (Block) had only three levels, below the recommended five levels for random effects (Gelman & Hill, 2007). We analyzed insect herbivory and powdery mildew at the tree level. Due to the rarity of leaf rollers and webbers, we aggregated herbivore abundance into three guilds for analysis: gallers, leaf miners, and leaf manipulators (skeletonizers, rollers, and webbers). Numbers of each herbivore group were summed over the 10 primary shoots sampled per tree to produce tree level totals. For powdery mildew, we averaged infection scores across the 10 shoots surveyed per tree.

Data from different sites and years were analyzed separately for the three insect herbivore guilds and powdery mildew. We analyzed the 2 years of data from Hucking separately as tree height was measured only in 2017, so we ran separate models for the 2 years with slightly different explanatory variables. Data from the two sites were analyzed separately since the origin of the local and Italian provenances differed between them (with only the French provenance in common between sites).

For powdery mildew infection and for each herbivore guild, we therefore produced three models: Hucking 2016, Hucking 2017, and Hartshorne 2017 (Table 1). Model explanatory variables were as follows: tree provenance, tree diversity (diversity treatment), tree vigor (individual tree height and average shoot length), and tree apparency. The full model for each foliar group at each site and year included diversity treatment, provenance and block as categorical predictors, and tree height and shoot length as continuous predictors (Table 1). Models of powdery mildew infection included lammas shoot length as a predictor, herbivore abundance models included primary shoot length as a predictor. Full models included all two‐way interactions between provenance, treatment, and block, and two‐way interactions between provenance and shoot length (Table 1). Prior to model fitting, we visually inspected continuous explanatory variables (tree height, shoot length) for linearity and normality, applying log transformations where necessary, and included quadratic terms in our models to account for nonlinear relationships when required (Table 1). We inspected multicollinearity between model variables using variance inflation factors (VIFs) calculated using the corvif() function R (Zuur, 2009). No VIF observed was >5, the suggested cut off point for collinearity between variables interfering with model inference (Zuur et al., 2009).

**Table 1:** An overview GLMs of oak powdery mildew infection and insect herbivore abundance, showing all explanatory variables included in starting models

Model response variable	Model explanatory variables	Model type
Oak Powdery Mildew (Hucking 2016)	Provenance [F]; Diversity [F]; Block [F]; Lammas Shoot Length [C]; Galler Abundance [C]; Leaf Miner Abundance [C]; Leaf Manipulator Abundance [C]; Provenance:Diversity [I]; Provenance:Block [I]; Diversity:Block [I]; Provenance:Lammas Shoot Length [I]	Gaussian GLM, log link
Oak Powdery Mildew (Hucking 2017)	Provenance [F]; Diversity [F]; Block [F]; Tree Height [C]; (Tree Height)^2^ [C]; Lammas Shoot Length [C]; Galler Abundance [C]; Leaf Miner Abundance [C]; Leaf Manipulator Abundance [C]; Provenance:Diversity [I]; Provenance:Block [I]; Diversity:Block [I]; Provenance:Lammas Shoot Length [I]	Gaussian GLM, log link
Oak Powdery Mildew (Hartshorne 2017)	Provenance [F]; Diversity [F]; Block [F]; Tree Height [C]; Lammas Shoot Length [C]; Galler Abundance [C]; Leaf Miner Abundance [C]; Leaf Manipulator Abundance [C]; Provenance:Diversity [I]; Provenance:Block [I]; Diversity:Block [I]; Provenance:Lammas Shoot Length [I]	Gaussian GLM, log link
Galler Abundance (Hucking 2016)	Provenance [F]; Diversity [F]; Block [F]; Primary Shoot Length [C]; Provenance:Diversity [I]; Provenance:Block [I]; Diversity:Block [I]; Provenance:Primary Shoot Length [I]	Negative binomial GLM, log link
Galler Abundance (Hucking 2017)	Provenance [F]; Diversity [F]; Block [F]; Tree Height [C]; Primary Shoot Length [C]; Provenance:Diversity [I]; Provenance:Block [I]; Diversity:Block [I]; Provenance:Primary Shoot Length [I]	Negative binomial GLM, log link
Galler Abundance (Hartshorne 2017)	Provenance [F]; Diversity [F]; Block [F]; Tree Height [C]; Primary Shoot Length [C]; Provenance:Diversity [I]; Provenance:Block [I]; Diversity:Block [I]; Provenance:Primary Shoot Length [I]	Negative binomial GLM, log link
Leaf Miner Abundance (Hucking 2016)	Provenance [F]; Diversity [F]; Block [F]; Primary Shoot Length [C]; Provenance:Diversity [I]; Provenance:Block [I]; Diversity:Block [I]; Provenance:Primary Shoot Length [I]	Negative binomial GLM, log link
Leaf Miner Abundance (Hucking 2017)	Provenance [F]; Diversity [F]; Block [F]; Tree Height [C]; Primary Shoot Length [C]; Provenance:Diversity [I]; Provenance:Block [I]; Diversity:Block [I]; Provenance:Primary Shoot Length [I]	Negative binomial GLM, log link
Leaf Miner Abundance (Hartshorne 2017)	Provenance [F]; Diversity [F]; Block [F]; Tree Height [C]; Primary Shoot Length [C]; Provenance:Diversity [I]; Provenance:Block [I]; Diversity:Block [I]; Provenance:Primary Shoot Length [I]	Poisson GLM, log link
Leaf Manipulator Abundance (Hucking 2016)	Provenance [F]; Diversity [F]; Block [F]; Primary Shoot Length [C]; Provenance:Diversity [I]; Provenance:Block [I]; Diversity:Block [I]; Provenance:Primary Shoot Length [I]	Negative binomial GLM, log link
Leaf Manipulator Abundance (Hucking 2017)	Provenance [F]; Diversity [F]; Block [F]; Tree Height [C]; Primary Shoot Length [C]; Provenance:Diversity [I]; Provenance:Block [I]; Diversity:Block [I]; Provenance:Primary Shoot Length [I]	Negative binomial GLM, log link
Leaf Manipulator Abundance (Hartshorne 2017)	Provenance [F]; Diversity [F]; Block [F]; Tree Height [C]; Primary Shoot Length [C]; Provenance:Diversity [I]; Provenance:Block [I]; Diversity:Block [I]; Provenance:Primary Shoot Length [I]	Negative binomial GLM, log link

Note

In the explanatory variables column, letters in brackets [] refer to the type of variable: F = Factor; C = Continuous; I = Interaction.

To test hypothesis 6, that high herbivory on primary growth may promote powdery mildew infection on lammas growth, for powdery mildew models, we included the log‐transformed abundance of gallers, leaf miners, and leaf manipulators as additional predictors.

Herbivore counts in all guilds were highly overdispersed and were analyzed using negative binomial GLMs with log link function, except for a Poisson GLM with log link for leaf miners at Hartshorne in 2017, where data was not overdispersed (Pearson's dispersion statistic = 1.04). For powdery mildew, we used a Gaussian GLM with log link function in order to constrain the response variable as positive but retain the normality and independence of residuals.

We carried out model simplification by comparing pairs of nested models using likelihood ratio testing, starting with removal of nonsignificant interaction terms, to achieve minimal adequate models retaining only significant explanatory variables. We used *F* tests for GLMs with Gaussian errors, and chi‐squared tests for GLMs negative binomial and Poisson errors (Crawley, 2007).

To test hypothesis 5, that the response to tree height by insect herbivores and powdery mildew was driven by tree apparency rather than just tree vigor, for models of 2017 data, we substituted tree apparency into minimal adequate models post hoc as a predictor instead of tree height. We compared the likelihood ratio test scores when either predictor was removed from the model, to assess which was the single best predictor of insect herbivory and powdery mildew. Although the VIF of neither apparency nor tree height was >5 at Hucking the two were still strongly correlated so we did not include both in the same initial model (value of Pearson's pairwise correlation coefficient = 0.61 at Hucking, 0.26 at Hartshorne).

We also carried out post hoc pairwise comparisons of means to test for specific differences in insect herbivore abundance and powdery mildew infection between provenances and diversity treatments, using *z* tests (function glht() from the multcomp package in R). We computed comparisons between means of factor levels only if no significant interaction terms were retained that could interfere with statistical inference (Table S1). Rather than calculating all pairwise comparisons between means, we computed comparisons only where model summaries indicated significant differences were present, as the probability of finding that at least one comparison is significant increases with each test (Gelman, Hill, & Yajima, 2012). For the model of gallers at Hucking in 2017, the minimal adequate model retained a significant interaction between provenance and treatment, so we carried out a subset analysis as visual exploration of the model predictions indicated there may be consistent differences in gall abundance between diversity treatments dependent on provenance. We ran a separate GLM for the Italian provenance only, fitting treatment, block and the interaction between treatment and block as the only predictors (Table S1).

We visualized model residuals to check model assumptions were met. To visualize final model predictions, we used partial residual plots to show the effects of significant predictors on response variables (Faraway, 2005).

## 3 RESULTS

### 3.1 Differences in tree phenotypic traits between provenances

#### 3.1.1 Primary shoot length

At Hucking in 2016, Local and French trees had significantly longer primary shoots than Italian trees, but did not significantly differ from each other (Table S2, Figure S1A). At both sites in 2017, Local trees had longer primary shoots than both French and Italian trees (Table S2, Figure S1B,C). At Hucking, nonlocal provenances did not differ in primary shoot length in 2017, while at Hartshorne, Italian trees had significantly shorter primary shoots than French trees (Table S2, Figure S1B,C).

#### 3.1.2 Lammas shoot length

At Hucking in both years, lammas shoot length was similar for all provenances (Table S2, Figure S1D,E). At Hartshorne, French trees had the longest lammas shoots, followed by Italian trees, with Local trees having significantly lower lammas shoot length than both nonlocal provenances (Table S2, Figure S1F).

#### 3.1.3 Tree height

The three oak provenances were of comparable tree height at Hucking (Table S2, Figure S2). At Hartshorne, tree heights were also comparable apart from French trees which were significantly taller than Italian trees (Table S2, Figure S2).

#### 3.1.4 Lammas shoot availability

At both sites, French and Italian trees had significantly higher lammas shoot scores than Local trees, but nonlocal provenances did not differ significantly from each other (Table S2, Figure S3).

### 3.2 Oak insect herbivore abundance and powdery mildew infection

**Figure 3:**
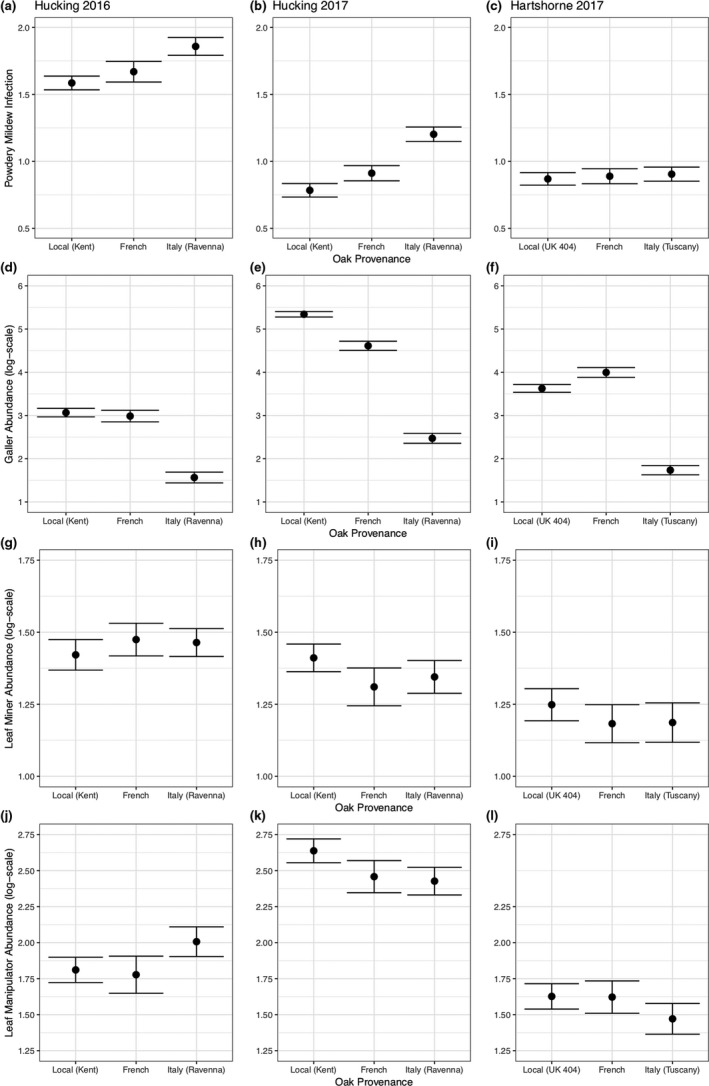
The impact of provenance identity on powdery mildew (a–c), gallers (d–f), leaf miners (g–i), and leaf manipulators (j–l). Plots show means of raw data (black circles) ± standard errors (error bars). The first column shows results at Hucking in 2016, the second column Hucking in 2017, the third column Hartshorne in 2017

**Figure 4:**
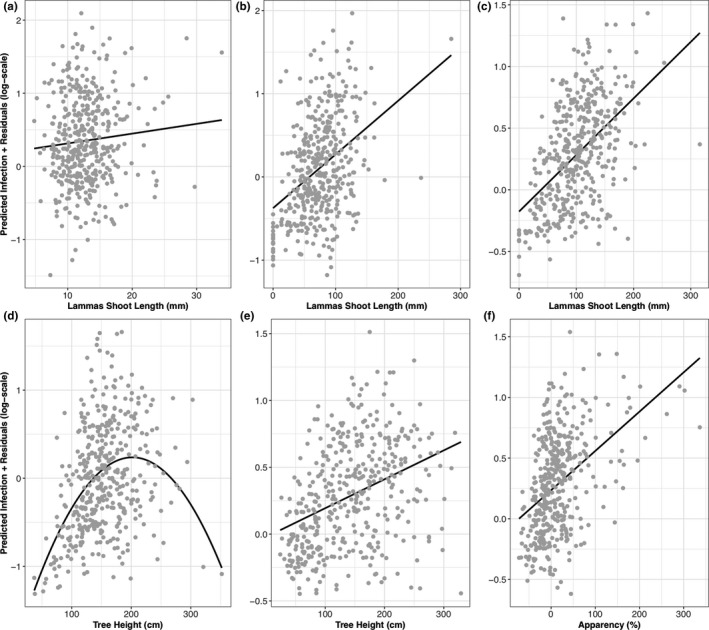
Partial residual plots showing the modeled impacts of lammas shoot length, tree height, and tree apparency on powdery mildew infection at Hucking and Hartshorne in 2016 and 2017. Model predictions are calculated by holding all other variables constant in the model. Gray dots show the predicted infection intensity of powdery mildew to which model residuals are added. The black regression line shows either a linear relationship (a–c, e,f) or quadratic (d) depending on which terms were significant in the final model. (a) Hucking in 2016; (b) Hucking in 2017; (c) Hartshorne in 2017; (d) Hucking in 2017; (e) Hartshorne in 2017; and (f) Hartshorne in 2017

Below, we describe impacts of provenance identity (Figure 3), tree diversity treatment Figures S5–S8), tree vigor (height and shoot length), and tree apparency (Figure 4) where these variables were retained in minimal adequate models (Tables 2 and 3).

**Table 2:** Summary of results of GLMs of oak powdery mildew. Results shown are likelihood ratio tests (*F* tests) for each variable retained in minimal adequate models

Model response variable	Year + Site	Explanatory variable	*df*	*F* value	*p* Value	Effect size and direction (if continuous)
Oak Powdery Mildew	2016 Hucking	Provenance	2	8.05	<0.001	***
		Diversity	6	/	/	/
		Block	2	/	/	/
		Lammas Shoot Length	1	6.57	0.011	+*
		Leaf Miner Abundance	1	5.05	0.025	+*
		Diversity: Block	12	8.74	<0.001	***
	2017 Hucking	Provenance	2	/	/	/
		Diversity	6	3.71	0.0014	**
		Block	2	4.26	0.015	*
		Lammas Shoot Length	1	/	/	/
		Tree Height	1	34.00	<0.001	+***
		Tree Height^2^	1	24.52	<0.001	−***
		Provenance : Lammas Shoot Length	2	3.74	0.025	*
	2017 Hartshorne	Provenance	2	/	/	/
		Diversity	6	4.65	<0.001	***
		Block	2	/	/	/
		Lammas Shoot Length	1	/	/	/
		Tree Height	1	5.09	0.025	+*
		Apparency	1	12.20	<0.001	+***
		Provenance: Block	4	4.91	<0.001	***
		Provenance: Lammas Shoot Length	2	3.92	0.021	*

Note

Stars denote level of significance in *F* tests (*p < 0.05; **p < 0.01, ***p < 0.001) with +/− indicating the direction of effect (positive or negative) for continuous variables. Note that for models with interaction effects, likelihood ratio tests cannot be calculated on the main effect without removing the interaction term from the model, so are not reported here (/ indicated within the relevant table column).

**Table 3:** Summary of results of GLMs of oak insect herbivores. Results shown are likelihood ratio tests (Chi‐squared tests) for each variable retained in minimal adequate models

Model response variable	Year + Site	Explanatory variable	*df*	χ^2^ value	*p* Value	Effect size and direction (if continuous)
Gallers	2016 Hucking	Provenance	2	64.80	<0.001	***
		Diversity	1	/	/	/
		Block	2	/	/	/
		Primary shoot length	1	27.81	<0.001	+***
		Diversity: Block	12	31.25	0.0018	**
	2017 Hucking	Provenance	2	/	/	/
		Diversity	6	/	/	/
		Block	2	21.33	<0.001	***
		Primary shoot length	1	37.28	<0.001	+***
		Tree height	1	27.74	<0.001	+***
		Provenance: Diversity	7	35.15	<0.001	***
	2017 Hartshorne	Provenance	2	/	/	/
		Diversity	6	/	/	/
		Block	2	/	/	/
		Primary shoot length	1	13.52	<0.001	+***
		Apparency	1	42.31	<0.001	+***
		Provenance: Diversity	7	20.23	0.0051	**
		Diversity: Block	12	51.55	<0.001	***
Leaf miners	2016 Hucking	Diversity	6	14.98	0.020	*
		Primary shoot length	1	15.56	<0.001	+***
		Block	2	12.75	0.0017	**
	2017 Hucking	Diversity	6	/	/	/
		Block	2	/	/	/
		Primary shoot length	1	20.11	<0.001	+***
		Apparency	1	8.77	0.0031	+**
		Diversity: Block	12	42.35	<0.001	***
	2017 Hartshorne	Provenance	2	6.28	0.043	*
		Diversity	6	/	/	/
		Block	2	/	/	/
		Primary shoot length	1	31.18	<0.001	+***
		Apparency	1	53.33	<0.001	+***
		Diversity: Block	12	29.12	0.0022	**
Leaf manipulators	2016 Hucking	Primary shoot length	1	11.64	<0.001	+***
	2017 Hucking	Provenance	2	6.62	0.037	*
		Diversity	6	/	/	/
		Block	2	/	/	/
		Primary shoot length	1	15.18	<0.001	+***
		Tree height	1	19.73	<0.001	+***
		Diversity: Block	12	32.23	0.0013	**
	2017 Hartshorne	Diversity	6	21.39	0.0016	**
		Block	2	103.48	<0.001	***
		Primary shoot length	1	6.28	0.012	+*
		Apparency	1	9.49	0.0021	+**

Note

Stars denote level of significance in Chi‐squared tests (*p < 0.05; **p < 0.01, ***p < 0.001) with +/− indicating the direction of effect (positive or negative) for continuous variables. Note that for models with interaction effects, likelihood ratio tests cannot be calculated on the main effect without removing the interaction term from the model, so are not reported here (/ indicated within the relevant table column).

#### 3.2.1 Impacts of provenance identity

##### Powdery mildew

Italian provenance trees had higher powdery mildew infection compared to Local trees in both years at Hucking (*z* = −4.01, *p* ≤ 0.001 in 2016; *z* = −3. 83, *p* ≤ 0.001 in 2017), while there was no difference between Local and French trees. Italian trees had higher infection than French trees in 2016 (*z* = −2.64, *p* = 0.0083), but not in 2017 (*z* = −1.598, *p* = 0.11). In contrast, at Hartshorne, we found no consistent differences in powdery mildew infection between provenances (Figure 3C and Figure S5).

##### Gallers

Gallers showed the most consistent response to provenance among insect guilds. Abundance was lowest on Italian trees across sites and years (Figure 3d–f). At Hucking in 2016, Italian provenance trees had significantly fewer galls compared to Local (*z* = 8.42, *p* < 0.001) and French (*z* = 5.164, *p* < 0.001) trees, while Local and French trees did not significantly differ (*z* = 1.68, *p* = 0.094). At Hucking in 2017, Local trees had the highest gall abundance, followed by French (compared to Local, in monoculture: *z* = −2.79, *p* = 0.0052) and Italian trees (compared to Local, in monoculture: *z* = −12.57, *p* < 0.001). French trees also had significantly more galls than Italian trees (in monoculture: *z* = −9.78, *p* < 0.001). However, the magnitude of differences between provenances varied between treatments (significant provenance by treatment interaction, Figure S6, Table 3). At Hartshorne, gall abundance was significantly higher on French trees compared to Local trees (in monoculture: *z* = 3.66, *p* < 0.001), while Italian trees had lower gall abundance than both Local (in monoculture: *z* = −7.82, *p* < 0.001) and French trees (in monoculture: *z* = −11.84, *p* < 0.001). However, like at Hucking in 2017, the magnitude of provenance differences varied between treatments (Figure S7, Table 3).

##### Leaf miners

Provenance was only a significant predictor of mine abundance at Hartshorne, where differences were small yet significant (Figure 3g–i). Both Local and French trees had higher mine abundance than Italian trees (Local vs. Italian: *z* = 2.68, *p* = 0.0075; French vs. Italian: *z* = 2.36, *p* = 0.018), but were not significantly different from each other (*z* = 0.055, *p* = 0.96).

##### Leaf manipulators

Provenances did not differ in leaf manipulator abundance at Hucking in 2016, while in 2017, Local trees had a higher abundance of leaf manipulators compared to Italian trees (*z* = 2.59, *p* = 0.0097) but not French trees (*z* = 0.80, *p* = 0.42), and French and Italian trees did not significantly differ (*z* = −1.44, *p* = 0.15). There were no significant differences between provenances at Hartshorne (Figure 3j–l).

#### 3.2.2 Impacts of tree diversity treatment

##### Overall patterns

The influence of tree diversity treatment on insect herbivory and powdery mildew differed between blocks within sites (significant interaction between treatment and block retained in most models, Tables 2 and 3). We also did not see consistent patterns when comparing between sites, or between survey years at Hucking. Below, we report differences between specific treatment levels only where they were consistent between blocks at each site.

##### Powdery mildew

At Hucking treatment effects were variable between blocks in 2016, while in 2017, powdery mildew infection was significantly lower in mixed species, mixed provenance plots compared to all provenance monocultures (*z* = 2.78, *p* = 0.0054). Powdery mildew infection was also lower on all trees in 50:50 and 75:25 mixtures of Local and French provenance trees compared to provenance monocultures (*z* = 1.98, *p* = 0.048 and *z* = 3.08, *p* = 0.0021 respectively). At Hartshorne the only consistent difference between treatments was that powdery mildew infection was significantly lower across provenances in 33:33:33 mixtures compared to all other plots (*z* = 2.39, *p* = 0.017).

##### Gallers

At Hucking in 2016, response to treatments was variable between blocks (Table 3). At Hucking in 2017, response varied according to provenance within treatments (Figure S6). Italian trees had higher gall abundance when mixed with Local trees, compared to monocultures of Italian trees (Figure S6). This was confirmed by a subset analysis of Italian trees only; Italian trees had higher gall abundance in both 50:50 mixes with Local trees compared to Italian monocultures (*z* = 2.36, *p* = 0.019) and in 33:33:33 provenance mixtures compared to Italian monocultures (*z* = 3.89, *p* ≤ 0.001). However, this associational susceptibility when mixed with a more susceptible provenance was only observed in 2017 at Hucking. Despite the same patterns of provenance susceptibility occurring in 2016, Italian trees were not more susceptible in mixes with Local trees. At Hartshorne, there were no consistent treatment differences across blocks, apart from a lower abundance of gallers on both provenances in mixed species, mixed provenance plots compared to others (Table 3 and Figure S7).

##### Leaf miners

At Hucking in 2016, abundance of mines was significantly lower on both Local and Italian provenances in mixed species, mixed provenance plots compared to all other plots, (*z* = 2.80, *p* = 0.0051). There were no other consistent treatment effects in 2016, and in 2017, the response to treatment differed between blocks at Hucking. At Hartshorne, the only consistent difference between blocks was lower abundance in mixed species, mixed provenance plots compared to provenance monocultures (Figure S8).

##### Leaf manipulators

There was no significant treatment effect on the abundance of leaf manipulators at Hucking in 2016 and no consistent response to treatment across blocks in 2017. At Hartshorne, leaf manipulator abundance was significantly higher in 75:25 mixtures of Local and Italian trees compared to provenance monocultures (*z* = −2.40, *p* = 0.016). Leaf manipulator abundance was also significantly lower in 33:33:33 plots, compared to provenance monocultures (*z* = 2.00, *p* = 0.046).

#### 3.2.3 Impacts of plant vigor

Both tree height and shoot length were significant positive predictors in all models, apart from leaf manipulators at Hartshorne, where only primary shoot length, not tree height, was a significant predictor (Table 3). At Hucking in 2017, the quadratic term for tree height was retained as a significant predictor (Figure 4d) suggesting a nonlinear relationship between tree height and powdery mildew infection with a “peak” infection level followed by a decrease at taller tree heights. This term remained significant even when outliers were removed (Figure S9). There were significant interactions between provenance and lammas shoot length on powdery mildew at both Hucking and Hartshorne in 2017 (Table 2). The strength of the relationship between powdery mildew infection intensity and lammas shoot length differed between provenances, though remained positive across provenances.

#### 3.2.4 Impacts of plant apparency

At Hucking, tree height (and its quadratic term) was a better predictor of powdery mildew infection than apparency (Table 2). At Hartshorne, apparency was a better single predictor of powdery mildew than tree height (Figure 4e,f) though both remained significant when included in a single model (Table 2). For gallers, tree height was a better predictor of gall abundance than apparency at Hucking, while at Hartshorne, apparency was a better predictor (Table 3). For leaf miners, apparency was a better predictor than tree height at both sites (Table 3). For leaf manipulators, at Hucking tree height was a better predictor than apparency (Table 3). At Hartshorne, the main effect of tree height was nonsignificant; however, apparency was a significant predictor.

Plant apparency was not systematically linked to diversity treatment at either site, apart from mixed species plots at Hartshorne in 2017, where oak apparency was higher than other treatments, due to poor growth of other planted tree species (Figure S4).

#### 3.2.5 Insect herbivore abundance as a predictor of oak powdery mildew infection

**Figure 5:**
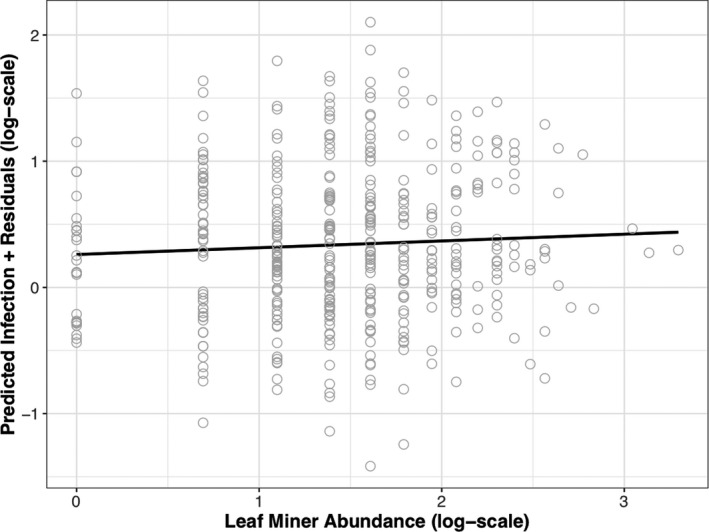
Partial residual plot showing the conditional impact of leaf miner abundance on powdery mildew infection at Hucking (2016), when all other variables are held constant in the model. Model predictions are calculated by holding all other variables constant in the model. Gray dots show the predicted infection intensity of powdery mildew to which model residuals are added, plus black regression line. Leaf miner abundance was a significant positive predictor of powdery mildew infection (*F*_(1,395)_ = 5.05, *p* = 0.0252)

At Hucking in 2016, there was a significant positive correlation between leaf miner abundance on primary growth, and mildew infection on lammas growth (Figure 5). However, this result was not consistent between years, as in 2017, none of the insect herbivore groups were significant predictors of mildew infection (Table 2).

## 4 DISCUSSION

We found inconsistent impacts of admixing species and provenances on oak insect herbivory and powdery mildew between years and sites. We did not find consistent evidence to support local adaptation by foliar organisms to local tree genotypes, compared to regionally climate‐matched tree provenances, though provenance identity impacted both gallers and powdery mildew. We found strong support for the plant vigor hypothesis across insect herbivore groups, and plant vigor also significantly predicted powdery mildew infection. It is likely that both individual plant vigor and growing context (plant apparency) jointly influence foliar damage organisms on oak.

### 4.1 Inconsistent effects of tree species diversity

Previous studies have found that admixing oak with other tree species can have significant associational effects on insect herbivore and mildew damage (Castagneyrol et al., 2013; Hantsch et al., 2014; Moore et al., 1991). Based on these previous findings, in our study, we expected that both insect herbivore abundance and powdery mildew infection would decrease in mixed species plots compared to all others across sites and years, due to increased dilution of susceptible oaks by nonhost species. Although associational resistance was observed in some comparisons between mixed species plots and oak monocultures, no group of foliar organisms was consistently reduced across sites and years in mixed species plots. Inconsistent tree diversity effects on insect herbivores between years within the same diversity experiment have also been observed in other studies (Castagneyrol et al., 2017, 2013). Like Climate Match, most tree diversity experiments used in such studies are still at the establishment stage (Verheyen et al., 2016). Variation between survey years, and between trial sites, could have included ontological changes to tree physiology over time, and contrasting edaphic conditions differentially impacting tree growth and apparency relative to understorey vegetation and surrounding trees. These temporal and spatial sources of variation between years and geographic locations could have outweighed potential dilution effects on pests and pathogens caused by tree diversity. Moreover, differing local climatic conditions between sites and years could have impacted plot microclimate and tree traits, altering diversity effects on insect herbivores and powdery mildew (Castagneyrol, Jactel, et al., 2018; Walter et al., 2012). Our results therefore emphasize the potential importance of environmental context modulating the outcome of associational effects on pests and pathogens (Castagneyrol, Jactel, et al., 2018).

### 4.2 Provenance matters

French and Italian provenances planted at the Climate Match experimental sites were selected to be adapted to UK climates 50 and 80 years in the future, respectively (Broadmeadow et al., 2005). We found that provenance identity impacted insect herbivory and powdery mildew, but with contrasting results between groups. Contrary to the prediction that insect herbivores and mildew will be adapted to exploit local host tree phenotypes, nonlocal provenances were not always less susceptible to either insect herbivory or powdery mildew infection. Although Italian provenance trees consistently escaped herbivory by gallers across sites and years compared to Local and French provenances, they did not escape herbivory by other insect guilds or powdery mildew infection. In fact, in both years, powdery mildew infection at one site (Hucking) was higher on Italian provenance trees compared to local provenance. This contrasts with a reciprocal transplant study which found evidence for local adaptation by mildew populations to individual oaks (Roslin, Laine, & Gripenberg, 2007). However, this study took place at a different spatial scale, with alternative host trees sourced from the same island population, rather than different geographic regions as in our study. Cynipid gallwasp larvae develop inside oak tissue which they physiologically manipulate to form a gall which provides both physical protection and a food source (Harper, Schonrogge, Lim, Francis, & Lichtenstein, 2004; Stone & Schönrogge, 2003; Stone, Schönrogge, Atkinson, Bellido, & Pujade‐Villar, 2001). By comparison, leaf miners and manipulators do not exercise the same level of control over the plant tissue on which they feed, and these guilds were therefore expected to demonstrate a stronger response to variation in plant tissue quality across provenances (Huguet, Stone, & Body, 2016; Sopow, Shorthouse, Strong, & Quiring, 2003). Contrary to this, we found that gallwasps showed a stronger response to provenance than both leaf miners and leaf manipulators.

Phenological differences between provenances may explain the contrasting responses of gallwasps and powdery mildew infection to tree provenance. Italian provenance trees had more lammas shoots compared to Local and French trees by the same date in July (Figure S3). Kleinschmit (1993) suggested that oak provenances from more southerly latitudes had earlier emergence of lammas shoots and thus higher cross‐continental susceptibility to powdery mildew. Although our findings partially support this hypothesis, at Hartshorne, Italian and French provenances were not more susceptible to powdery mildew, despite showing earlier emergence of lammas shoots compared to Local trees (Figure S3). Gallwasps require host tissues to be at the correct physiological age for successful gall induction (Weis, 1988), and local adaptation to bud burst has been observed in gallwasp species (Egan & Ott, 2007). Sinclair et al. (2015) found variation in gallwasp abundance across sessile oak (*Q*. petraea) provenances differing in the timing of spring budburst. It is possible that phenological asynchrony of Italian provenances with local galler populations may explain why Italian trees in our study had lowest gall abundance across sites and years. Some galler groups are also known to respond positively to plant module size (Flaherty & Quiring, 2008; Kopelke, Amendt, & Schönrogge, 2003). We found differential resource allocation to plant modules across provenances; local trees invested in longer primary shoots, nonlocal provenances produced more/longer lammas growth (Figures S1 and S3). These differences may partly explain variation in provenance susceptibility to insect herbivores and mildew. However, as is usual for correlational studies, other nonmeasured differences between provenances could also contribute to variation in herbivory and mildew infection, such as leaf chemistry (foliar nutrition) and defense (Pearse, 2011). As local and nonlocal provenances did not consistently differ in herbivory or mildew, this could explain the lack of consistent associational effects seen in mixed provenance plots compared to provenance monocultures.

### 4.3 Positive impacts of tree vigor and apparency

Tree vigor and apparency were consistent predictors of insect herbivory and powdery mildew infection. More vigorous trees (taller, with longer lammas shoots, Figure 4), and more apparent trees (Figure 4) had higher levels of powdery mildew infection at both sites. As biotrophic pathogens, powdery mildews may respond positively to vigorous hosts with a higher photosynthetic rate (Schnathorst, 1965). To our knowledge, our study is the first to show a positive association between oak powdery mildew and tree vigor, as well as tree apparency. Our results are also consistent with the plant vigor hypothesis for insect herbivores, which predicts higher insect abundance on more vigorous hosts, potentially due to trade‐offs between growth and defense (Price, 1991). But, tree apparency was a better predictor than tree height in most models of insect herbivory, and in one case (leaf manipulator abundance at Hartshorne in 2017) apparency was a significant predictor while tree height was not.

Unlike previous work, tree apparency was not systematically linked to diversity treatment in these trials (Castagneyrol et al., 2013)**.** Here, we found that taller trees experienced higher herbivory and mildew, especially when growing in the context of less vigorous surrounding trees. For insect herbivores, apparency may determine host searching efficiency, as has been suggested previously for the pine processionary moth (Régolini et al., 2014). For powdery mildew, a passive, wind‐dispersed pathogen (Marçais & Desprez‐Loustau, 2014), spore interception may be more efficient when hosts are taller and therefore more prominent in the landscape.

### 4.4 No clear association between insects and powdery mildew

We did not find a consistent association between insect herbivores and powdery mildew, although at one site in 1 year (Hucking in 2016) leaf miner abundance on primary shoots was positively correlated with powdery mildew infection on lammas growth. This suggests either that leaf miners and powdery mildew preferred the same trees, or that there is a direct interaction between the two groups. Previous work has suggested that early season herbivore pressure can promote subsequent bud flushes, increasing the amount of leaf material susceptible to mildew infection (Marçais & Desprez‐Loustau, 2014). Alternatively, an interaction between damage agents could occur through cross‐talk between plant defense signaling pathways (Schultz, Appel, Ferrieri, & Arnold, 2013). Meta‐analysis has shown that herbivores inducing the jasmonic acid (JA) pathway (including leaf miners) have an antagonistic effect on subsequent herbivores inducing the salicylic acid (SA) pathway, which is also induced by biotrophic pathogens (Moreira et al., 2018). Understanding interactions between insects and pathogens is important as many plant health problems, such as oak decline, can have multiple causative agents (Denman et al., 2018; Thomas, 2008). More work is required to establish the strength and consistency of the interaction between insects and powdery mildew, and to establish potential underlying mechanisms in terms of plant phenology and defense.

## 5 CONCLUSION

We found that the effects of both tree species and genetic diversity were variable, suggesting that forest diversity effects on pests and pathogens can be unpredictable across space and time. In addition, we show that individual tree vigor and growth relative to surrounding trees can drive colonization of tree hosts by herbivores and pathogens, providing compelling support for both the plant vigor and plant apparency hypotheses.

## CONFLICT OF INTEREST

None declared.

## AUTHOR CONTRIBUTIONS

MG, KS, NB, and EF devised the study. EF carried out the field assessments with other trained observers. EF analyzed the data with input and advice from AH, MG, and KS. EF wrote the manuscript. All authors commented on previous and final versions of the manuscript.

## Data Availability

Data for this study are available for download from the NERC Environmental Information Data Centre (https://doi.org/10.5285/cbccb101-c877-4e43-ac70-e8a852b51f07).
