# Supplementary material for: Individual tree traits shape insect and disease damage on oak in a climate‐matching tree diversity experiment
Source: Ecol Evol. 2019 Jul 17;9(15):8524–40. doi: 10.1002/ece3.5357 (PMC6686283; doi:10.1002/ece3.5357)
Supplement: Supplementary file 1 [file ECE3-9-8524-s001.docx]

**INDIVIDUAL TREE TRAITS SHAPE INSECT AND DISEASE DAMAGE ON OAK IN A CLIMATE-MATCHING TREE DIVERSITY EXPERIMENT**

**Authors:**

Field, E.^1*^, Schönrogge, K.^2^, Barsoum, N.^3^, Hector, A.^1^ & Gibbs, M.^2^

**Authors affiliations:**

^1^ Department of Plant Sciences, South Parks Road, University of Oxford, Oxford, OX1 3RB, United Kingdom; ^2^Centre for Ecology & Hydrology, MacLean Building, Crowmarsh Gifford, Wallingford, OX10 8BB, United Kingdom; ^3^ Forest Research, Alice Holt Lodge, Farnham, Surrey, GU10, 4LH, United Kingdom

*Corresponding author: elsa.field@plants.ox.ac.uk

**-Supplementary material-**

Post Hoc Analysis

- Table S1: Explanation of the post hoc tests and/or analyses completed for each model.

Tree Traits

- Table S2: F tests for one-way ANOVAs testing the effect of provenance on tree phenotypic traits
- Figure S1: Differences in primary shoot length and lammas shoot length between provenances.
- Figure S2: Differences in tree height between provenances.
- Figure S3: Differences in lammas shoot availability between provenances.
- Figure S4: Boxplots showing differences in tree apparency and tree height between diversity treatments in the two sites.

Powdery Mildew and Foliar Insect Herbivores

- Table S3: Summary of the effects of diversity treatment on powdery mildew infection and insect herbivore abundance at each site and year.

Appendix: Partial Residual Plots to visualise significant predictors in minimal adequate models

Figure S5 - Partial residual plot showing the conditional effects of provenance and block on powdery mildew at Hartshorne in 2017

Figure S6 - Partial residual plot showing the conditional effects of provenance and treatment on gall abundance at Hucking in 2017

Figure S7 - Partial residual plot showing the conditional effects of provenance on gall abundance at Hartshorne in 2017

Figure S8 - Partial residual plot showing the conditional effects of treatment and block on leaf miner abundance at Hartshorne in 2017

Figure S9: Partial residual plot showing the impact of tree height on powdery mildew infection for an updated model where outliers are removed, Hucking 2017

Post Hoc Analyses

**Table S1: Explanation of the post hoc tests and/or analyses completed for each model.**

| **Model Response Variable** | **Retained Variables in Minimal Adequate Model** | **Model Type** | **Post Hoc Analyses Conducted** |
| --- | --- | --- | --- |
| Oak Powdery Mildew (Hucking 2016) | Provenance [F]; Diversity [F]; Block [F]; Lammas Shoot Length [C]; Leaf Miner Abundance [C]; Diversity:Block [I] | Gaussian GLM, log link | 1. Pairwise comparison of means between provenances (z tests) |
| Oak Powdery Mildew (Hucking 2017) | Provenance [F]; Diversity [F]; Block [F]; Lammas Shoot Length [C]; Tree Height [C]; Tree Height^2^ [C]; Provenance:Lammas Shoot Length [I] | Gaussian GLM, log link | 1. Apparency included post hoc 1) in addition to 2) instead of tree height variables, likelihood ratio test performed to compare significance 2. Pairwise comparison of means between provenances (z tests) 3. Pairwise comparison of means between diversity treatments (z tests) for:  - Mixed Species versus All Provenance Monocultures - 50:50 Provenance Mixtures versus All Provenance Monocultures - 75:25 Provenance Mixtures versus All Provenance Monocultures |
| Oak Powdery Mildew (Hartshorne 2017) | Provenance [F]; Diversity [F]; Block [F]; Lammas Shoot Length [C]; Tree Height [C]; Apparency [C]; Provenance:Block [I]; Provenance:Lammas Shoot Length [I] | Gaussian GLM, log link | 1. Apparency included post hoc 1) in addition to 2) instead of tree height, likelihood ratio test performed to compare significance 2. Pairwise comparison of means between diversity treatments (z tests) for:  - 33:33:33 Provenance Mixtures versus all other plots |
| Galler Abundance (Hucking 2016) | Provenance [F]; Diversity [F]; Block [F]; Primary Shoot Length [C]; Diversity:Block [I] | Negative binomial GLM, log link | 1. Pairwise comparison of means between provenances (z tests) |
| Galler Abundance (Hucking 2017) | Provenance [F]; Diversity [F]; Block [F]; Primary Shoot Length [C]; Tree Height [C]; Provenance:Diversity [I] | Negative binomial GLM, log link | 1. Apparency included post hoc 1) in addition to 2) instead of tree height, likelihood ratio test performed to compare significance 2. Pairwise comparison of means between provenances (z tests) 3. To test the outcome of the provenance:diversity interaction, reran the model for Italian provenance only. Pairwise comparisons of means performed to compare:  - 50:50 Provenance Mixes with Italian Monocultures - 33:33:33 Provenance Mixes with Italian Monocultures |
| Galler Abundance (Hartshorne 2017) | Provenance [F]; Diversity [F]; Block [F]; Primary Shoot Length [C]; Apparency [C]; Provenance:Diversity [I]; Diversity:Block [I] | Negative binomial GLM, log link | 1. Apparency included post hoc 1) in addition to 2) instead of tree height, likelihood ratio test performed to compare significance 2. Pairwise comparison of means between provenances (z tests) |
| Leaf Miner Abundance (Hucking 2016) | Diversity [F]; Block [F]; Primary Shoot Length [C] | Negative binomial GLM, log link | 1. Pairwise comparison of means (z tests) between Mixed Species, Mixed Provenance plots compared to all others |
| Leaf Miner Abundance (Hucking 2017) | Diversity [F]; Block [F]; Primary Shoot Length [C]; Apparency [C]; Diversity:Block [I] | Negative binomial GLM, log link | 1. Apparency included post hoc 1) in addition to 2) instead of tree height, likelihood ratio test performed to compare significance |
| Leaf Miner Abundance (Hartshorne 2017) | Provenance [F]; Diversity [F]; Block [F]; Primary Shoot Length [C]; Apparency [C]; Diversity:Block [I] | Poisson GLM, log link | 1. Apparency included post hoc 1) in addition to 2) instead of tree height, likelihood ratio test performed to compare significance 2. Pairwise comparison of means between provenances (z tests) |
| Leaf Manipulator Abundance (Hucking 2016) | Primary Shoot Length [C] | Negative binomial GLM, log link | None |
| Leaf Manipulator Abundance (Hucking 2017) | Provenance [F]; Diversity [F]; Block [F]; Primary Shoot Length [C]; Tree Height [C]; Diversity:Block [I] | Negative binomial GLM, log link | 1. Apparency included post hoc 1) in addition to 2) instead of tree height, likelihood ratio test performed to compare significance 2. Pairwise comparison of means between provenances (z tests) |
| Leaf Manipulator Abundance (Hartshorne 2017) | Diversity [F]; Block [F]; Primary Shoot Length [C]; Apparency [C] | Negative binomial GLM, log link | 1. Apparency included post hoc (tree height nonsignificant) 2. Pairwise comparison of means between diversity treatments (z tests) for:  - 75:25 Provenance Mixtures versus All Provenance Monocultures - 33:33:33 Provenance Mixtures versus All Provenance Monocultures |

Tree Traits

**Table S2: F tests for one-way ANOVAs testing the effect of provenance on tree phenotypic traits.** Significant results are in bold and italics. DF = degrees of freedom for F tests.

| Model Response Variable | Year + Site | F value | DF | P value |
| --- | --- | --- | --- | --- |
| Primary Shoot Length | 2016 Hucking | 6.47 | 2,417 | ***0.0017*** |
|  | 2017 Hucking | 39.67 | 2, 413 | ***< 0.001*** |
|  | 2017 Hartshorne | 47.80 | 2, 408 | ***< 0.001*** |
| Lammas Shoot Length | 2016 Hucking | 2.20 | 2,417 | 0.11 |
|  | 2017 Hucking | 2.32 | 2, 413 | 0.099 |
|  | 2017 Hartshorne | 12.01 | 2, 408 | ***< 0.001*** |
| Tree Height | 2017 Hucking | 1.33 | 2, 413 | 0.27 |
|  | 2017 Hartshorne | 2.24 | 2, 408 | 0.11 |
| Lammas Shoot Availability | 2017 Hucking | 23.81 | 2,135 | ***< 0.001*** |
|  | 2017 Hartshorne | 17.76 | 2,131 | ***< 0.001*** |


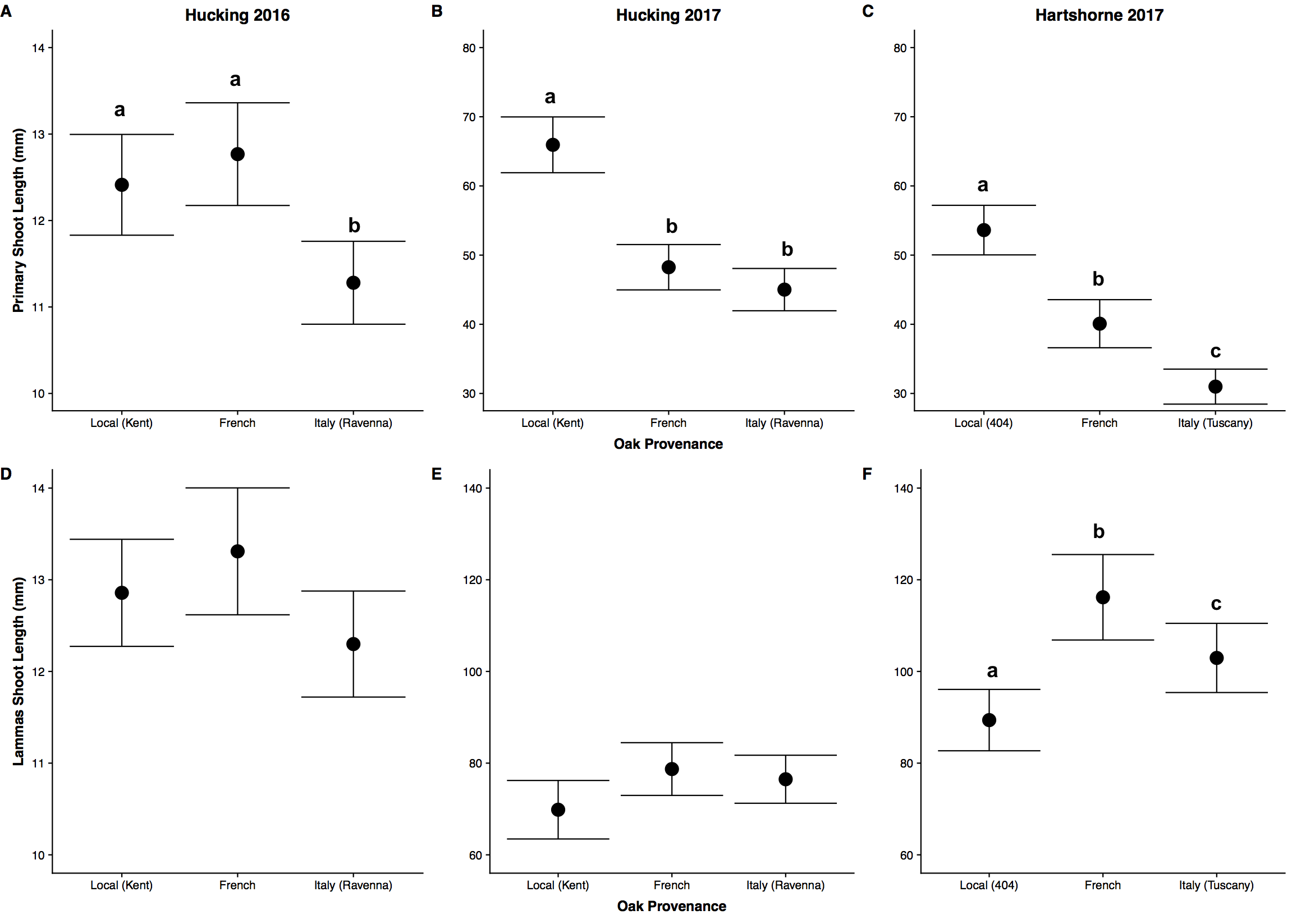


**Figure S1:** **Differences in primary shoot length (A-C) and lammas shoot length (D-F) between provenances.** A + D – Hucking 2016; B + E – Hucking 2017; C + F – Hartshorne 2017. Plotted are means (black circles) +/- 95% confidence intervals (error bars). Different letters above error bars indicate where significant differences between provenances occurred (p < 0.05) as calculated using one-way ANOVA.

**Figure S2:** **Differences in tree height between provenances**. A = Hucking 2017, B = Hartshorne 2017. Plotted are means (black circles) +/- 95% confidence intervals (error bars).


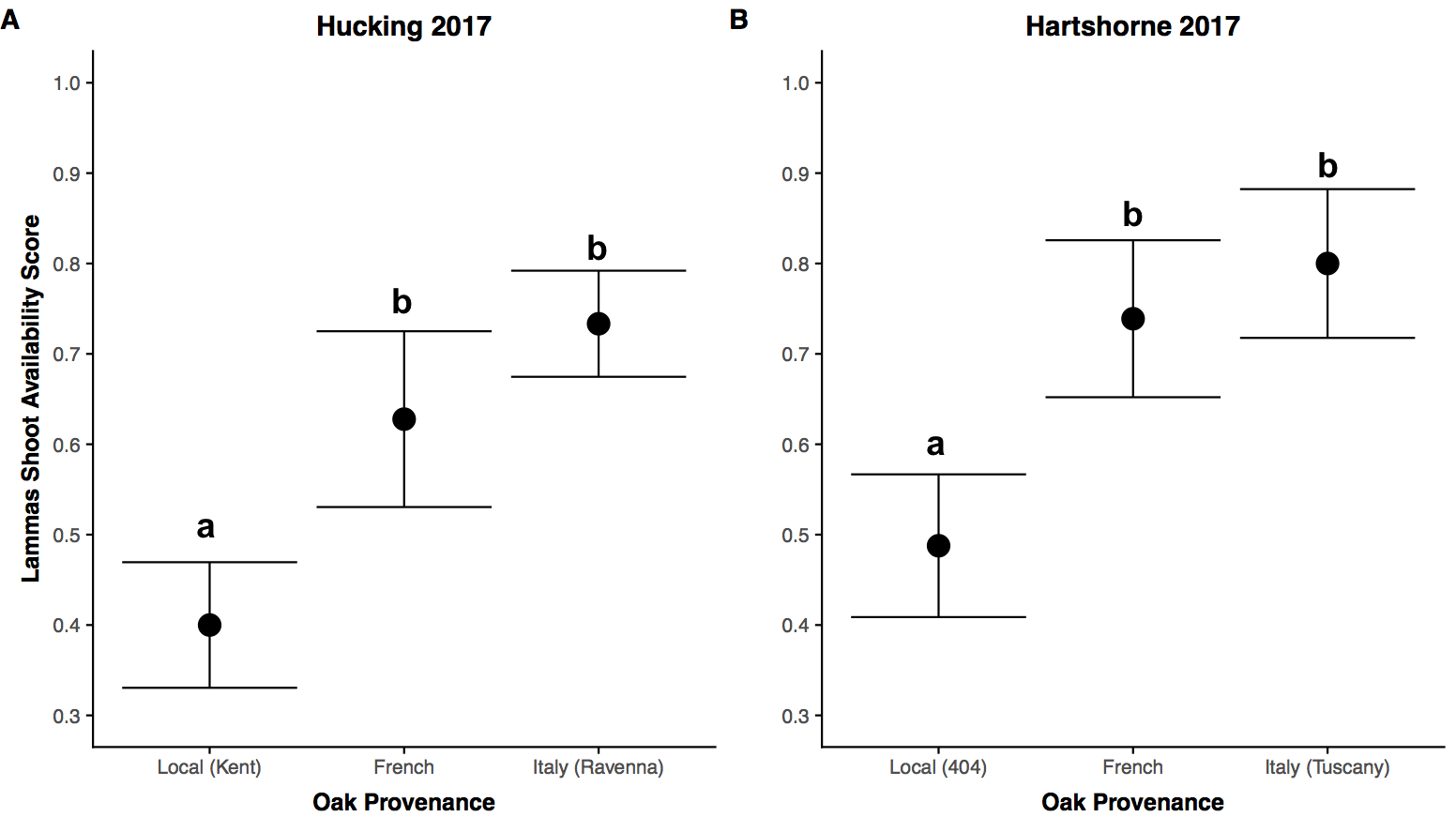


**Figure S3:** **Differences in lammas shoot availability between provenances.** A = Hucking 2017, B = Hartshorne 2017. Plotted are means (black circles) +/- 95% confidence intervals (error bars). Different letters above error bars indicate where significant differences between provenances occurred (p < 0.05) as calculated using one-way ANOVA.

**Figure S4:** **Boxplots showing differences in tree apparency and tree height between diversity treatments in the two sites.** A & C, Hucking 2017, B & D, Hartshorne 2017. Abbreviations for diversity treatments are as follows: Mono (provenance monoculture); MixSp (mixed species, mixed provenance); P1P2_50 (Local:French 50:50 mixture); P1P2_75 (Local:French 75:25 mixture), P1P3_50 (Local:Italian 50:50 mixture); P1P3_75 (Local:Italian 75:25), Thirty (33:33:33 mixture of Local, French and Italian provenances).

Powdery Mildew and Foliar Insect Herbivory

**Table S3:** **The effects of diversity treatment on powdery mildew infection and insect herbivore abundance at each site and year.** Numbers are plot level averages, so N = 3 for each treatment at each site and year (3 blocks per site with each treatment replicated once per block), except for Hartshorne, where N = 2 for mixed species plots as they are replicated in 2/3 blocks.

| Hucking 2016 | | | | | | | | |
| --- | --- | --- | --- | --- | --- | --- | --- | --- |
|  | MILDEW | | GALLERS | | MINERS | | MANIPULATORS | |
| TREATMENT | MEAN | SE | MEAN | SE | MEAN | SE | MEAN | SE |
| Provenance Monoculture | 1.80 | 0.28 | 31.10 | 8.02 | 4.43 | 0.51 | 11.87 | 0.33 |
| Mixed species | 1.77 | 0.13 | 19.83 | 5.11 | 3.11 | 0.31 | 13.61 | 1.65 |
| Local:French 50:50 | 1.99 | 0.18 | 37.39 | 5.64 | 4.74 | 0.53 | 16.22 | 3.31 |
| Local:French 75:25 | 1.31 | 0.17 | 37.53 | 5.28 | 3.53 | 0.35 | 9.31 | 1.05 |
| Local:Italian 50:50 | 1.54 | 0.33 | 19.62 | 5.05 | 4.98 | 0.90 | 10.93 | 1.61 |
| Local:Italian 75:25 | 1.65 | 0.38 | 11.72 | 2.17 | 4.34 | 0.23 | 12.36 | 2.98 |
| Local:French:Italian 33:33:33 | 1.72 | 0.29 | 33.01 | 3.50 | 3.85 | 0.29 | 12.08 | 4.92 |
| Hucking 2017 | | | | | | | | |
|  | MILDEW | | GALLERS | | MINERS | | MANIPULATORS | |
| TREATMENT | MEAN | SE | MEAN | SE | MEAN | SE | MEAN | SE |
| Provenance Monoculture | 1.13 | 0.08 | 152.88 | 41.77 | 4.24 | 0.85 | 19.40 | 4.63 |
| Mixed species | 0.84 | 0.07 | 122.07 | 54.43 | 3.98 | 0.57 | 22.54 | 7.48 |
| Local:French 50:50 | 0.84 | 0.06 | 282.98 | 27.78 | 4.56 | 0.78 | 16.24 | 2.73 |
| Local:French 75:25 | 0.56 | 0.06 | 220.77 | 22.64 | 2.84 | 0.23 | 17.37 | 1.24 |
| Local:Italian 50:50 | 1.13 | 0.11 | 150.63 | 20.56 | 3.30 | 0.68 | 20.98 | 4.24 |
| Local:Italian 75:25 | 0.98 | 0.13 | 125.65 | 33.82 | 3.21 | 0.42 | 17.33 | 0.90 |
| Local:French:Italian 33:33:33 | 1.06 | 0.08 | 135.29 | 10.79 | 3.92 | 0.46 | 19.17 | 0.44 |
| hartshorne 2017 | | | | | | | | |
|  | MILDEW | | GALLERS | | MINERS | | MANIPULATORS | |
| TREATMENT | MEAN | SE | MEAN | SE | MEAN | SE | MEAN | SE |
| Provenance Monoculture | 0.92 | 0.24 | 64.00 | 7.59 | 3.69 | 0.98 | 8.54 | 3.65 |
| Mixed species | 0.69 | 0.10 | 12.81 | 3.08 | 1.52 | 0.47 | 5.33 | 4.44 |
| Local:French 50:50 | 0.93 | 0.41 | 70.87 | 6.72 | 3.40 | 0.90 | 8.64 | 4.02 |
| Local:French 75:25 | 0.92 | 0.27 | 90.82 | 40.26 | 3.41 | 0.68 | 8.76 | 4.93 |
| Local:Italian 50:50 | 1.11 | 0.29 | 33.76 | 12.87 | 3.17 | 1.02 | 9.26 | 2.89 |
| Local:Italian 75:25 | 0.88 | 0.32 | 32.11 | 6.63 | 3.77 | 1.61 | 11.60 | 3.78 |
| Local:French:Italian 33:33:33 | 0.73 | 0.30 | 51.38 | 17.20 | 3.11 | 0.32 | 5.60 | 2.10 |

Appendix: Partial Residual Plots to visualise significant predictors in minimal adequate models

Partial residual plots are a type of model prediction plot that can be used for hypothesis testing, for instance, to clearly visualise the significance of multiple predictors when minimal adequate models contain interaction terms. In all plots one or two variables are varied while others are held constant at their mean value (continuous predictors) or a representative factor level (categorical predictors). Model residuals from the minimal adequate model (Table 2 and S1) are then added. For further discussion of partial residual plots, see Faraway (2005).

**Figure S5:** Partial residual plot showing the conditional effects of provenance and block on powdery mildew at Hartshorne in 2017. All other variables are held constant in the model (treatment = provenance monoculture). Different blocks (1-3) are shown in different panels. The plot shows the lack of consistency in the effect of provenance on powdery mildew infection when the effect of block is also considered. Plotted are means (open black circles), medians (horizontal line), interquartile range (box), upper and lower quartiles (whiskers) and outliers (black dots).

**Figure S6:** Partial residual plot showing the conditional effects of provenance and treatment on gall abundance at Hucking in 2017. All other variables are held constant in the model (block = 1). Plotted are medians (horizontal line), means (open circles), interquartile range (box), upper and lower quartiles (whiskers) and outliers (dots). Abbreviations for diversity treatments are as follows: Mono (provenance monoculture); MixSp (mixed species, mixed provenance); P1P2_50 (Local:French 50:50 mixture); P1P2_75 (Local:French 75:25 mixture), P1P3_50 (Local:Italian 50:50 mixture); P1P3_75 (Local:Italian 75:25), Thirty (33:33:33 mixture of Local, French and Italian provenances).

**BLOCK 1**

**BLOCK 2**

**Figure S7:** Partial residual plot showing the conditional effects of provenance and treatment on gall abundance at Hartshorne in 2017, in block 1 (top panel) and block 2 (bottom panel), as these two blocks contain the mixed species plots. All other variables are held constant in the model. Plotted are medians (horizontal line), means (open circles), interquartile range (box), upper and lower quartiles (whiskers) and outliers (dots). Differences between mixed species plots and provenance monocultures tested using z tests: for Local trees in Block 1: z = -2.64 p = 0.0084; for Italian trees in Block 1: z = -2.24 p = 0.025. Abbreviations for diversity treatments are as follows: Mono (provenance monoculture); MixSp (mixed species, mixed provenance); P1P2_50 (Local:French 50:50 mixture); P1P2_75 (Local:French 75:25 mixture), P1P3_50 (Local:Italian 50:50 mixture); P1P3_75 (Local:Italian 75:25), Thirty (33:33:33 mixture of Local, French and Italian provenances).

**Figure S8:** Partial residual plot showing the conditional effects of treatment and block on leaf miner abundance at Hartshorne in 2017. All other variables are held constant in the model (provenance = Local). Plotted are medians (horizontal line), means (open circle), interquartile range (box), upper and lower quartiles (whiskers) and outliers (dots). Abbreviations for diversity treatments are as follows: Mono (provenance monoculture); MixSp (mixed species, mixed provenance); P1P2_50 (Local:French 50:50 mixture); P1P2_75 (Local:French 75:25 mixture), P1P3_50 (Local:Italian 50:50 mixture); P1P3_75 (Local:Italian 75:25), Thirty (33:33:33 mixture of Local, French and Italian provenances).

 **Figure S9:** Partial residual plot showing the impact of tree height on powdery mildew infection for an updated model where outliers are removed, Hucking 2017. Two trees with heights > 310 cm (see figure 4D) have been removed. Grey dots show the predicted infection intensity of powdery mildew to which model residuals are added. The black regression line shows the modelled quadratic relationship between tree height and mildew infection. The quadratic term for tree height remains a significant predictor of mildew infection (F_(1,398)_ = 15.310, p = 0.000107).

**REFERENCES**

Faraway, J. (2005). *Extending the Linear Model with R: Generalized Linear, Mixed Effects and Nonparametric Regression Models.* CRC press.
